# Effects of Ion Irradiation and Temperature on Mechanical Properties of GaN Single Crystals under Nanoindentation

**DOI:** 10.3390/ma16247537

**Published:** 2023-12-06

**Authors:** Zhaohui Dong, Xiuyu Zhang, Jiling Li, Shengyuan Peng, Qiang Wan, Jianming Xue, Xin Yi

**Affiliations:** 1 School of Traffic Management, People’s Public Security University of China, Beijing 100038, China; zhdong@ppsuc.edu.cn; 2 Institute of Electronic Engineering, China Academy of Engineering Physics, Mianyang 621999, China; xiuyuzhang@pku.edu.cn; 3 Department of Mechanics and Engineering Science, College of Engineering, Peking University, Beijing 100871, China; lijingli@pku.edu.cn; 4 State Key Laboratory of Nuclear Physics and Technology, School of Physics, Peking University, Beijing 100871, China; 2101110314@pku.edu.cn; 5 Institute of Systems Engineering, China Academy of Engineering Physics, Mianyang 621999, China

**Keywords:** GaN single crystals, ion irradiation, high-temperature nanoindentation, hardness, mechanical properties

## Abstract

Understanding the impact of irradiation and temperature on the mechanical properties of GaN single crystals holds significant relevance for rational designs and applications of GaN-based transistors, lasers, and sensors. This study systematically investigates the influence of C-ion irradiation and temperature on pop-in events, hardness, Young’s modulus, and fracture behavior of GaN single crystals through nanoindentation experiments. In comparison with unirradiated GaN samples, the pop-in phenomenon for ion-irradiated GaN samples is associated with a larger critical indentation load, which decreases with increasing temperature. Both unirradiated and ion-irradiated GaN samples exhibit a decline in hardness with increasing indentation depth, while Young’s moduli do not exhibit a clear size effect. In addition, intrinsic hardness displays an inverse relationship with temperature, and ion-irradiated GaN single crystals exhibit greater intrinsic hardness than their unirradiated counterparts. Our analysis further underscores the significance of Peierls stress during indentation, with this stress decreasing as temperature rises. Examinations of optical micrographs of indentation-induced fractures demonstrate an irradiation embrittlement effect. This work provides valuable insights into the mechanical behavior of GaN single crystals under varying irradiation and temperature conditions.

## 1. Introduction

Gallium nitride (GaN), recognized for its wide bandgap, robust interatomic bonds, and exceptional thermal conductivity, stands as a prominent III–V semiconductor material [1]. It finds widespread applications in optoelectronics, high-temperature environments, and high-power devices, including light-emitting diodes, microwave power components, ultra-high-power switches, and laser diodes spanning from blue to the ultraviolet spectrum [2]. GaN crystals, renowned for their resilience to radiation damage [3], are integral to satellite systems, nuclear applications, and various extreme conditions where radiation resistance is paramount. Nevertheless, the microstructural damage incurred by high-energy particle irradiation on GaN crystals inevitably exerts an influence on their mechanical and physical characteristics, eventually impacting device longevity. Furthermore, GaN semiconductor devices frequently encounter elevated temperature during practical deployment. Thus, a comprehensive comprehension of the mechanical implications of radiation damage in this semiconductor material at high temperature can facilitate the advancement of high-performance and highly reliable GaN-based devices. This holds particular significance as device dimensions shrink to the microscale or below, necessitating a nanoscale understanding of GaN crystal mechanical properties under the combined effects of irradiation and temperature.

Given its low cost, low residual radioactivity, and high damage efficiency [4], ion irradiation experiments offer a suitable avenue for exploring the irradiation effects on GaN semiconductor devices. Nevertheless, the restricted depth of ion irradiation poses challenges when evaluating the macroscopic mechanical properties of irradiated materials using conventional mechanical testing methods like uniaxial tensile and compression tests. Instead, a range of small-scale mechanical testing techniques with high spatial resolution have been employed. Among these, nanoindentation stands out due to its relatively straightforward experimental setup and abundant, insightful data, making it a practical choice for studying the mechanical degradation of materials at the nanoscale [5,6,7].

At present, there is limited research on the mechanical properties of ion-irradiated GaN materials. While Kavouras et al. [8,9] explored the effects of Si^+^, Mg^+^, O^+^, and N^+^ ion implantation on the mechanical properties of epitaxially grown GaN samples using microhardness measurements, their findings were somewhat coarse, as they did not consider the plastic zone under the indenter extending from the ion-irradiated layer to the unirradiated layer. Some researchers have examined the mechanical properties of GaN materials after ion irradiation through experimental nanoindentation. For instance, Kucheyev et al. [10] delved into the pop-in event, hardness, and Young’s modulus of wurtzite GaN films modified by 2 MeV 197Au^+^ ion bombardment by nanoindentation with a spherical indenter. Jian et al. [11] studied the hardness and indentation size effects with increasing In concentration in the In_x_Ga_1−x_N films. However, they only showed results on the indentation size effect; mechanistic discussions were not provided. Eve et al. [12] investigated the pile-up, hardness, and Young’s modulus of GaN films irradiated with swift heavy uranium ions. More recently, our research explored the statistics of pop-in events of GaN single crystals irradiated by 3 MeV C^+^ ions, identifying a linear relationship between the regression slop as a micromechanical characterization and the hardness [13]. However, these studies have yet to address the influence of elevated temperature on the mechanical properties of ion-irradiated GaN. 

While separate studies have examined the mechanical properties of GaN materials at elevated temperature, the mutual effects of ion irradiation and high temperature on GaN’s mechanical properties remain unexplored. Notably, the works by Yonenage et al. [14], Lu et al. [15], and Wheeler et al. [16] have contributed insights into GaN’s mechanical behavior under varying conditions, but none have specifically investigated the combined effects of ion irradiation and elevated temperature. In addition, molecular dynamics (MD) simulations have been performed to study the mechanism of plastic deformation in irradiation GaN [17] and temperature effect on the mechanical response of c-plane monocrystalline GaN during nanoindentation [18]. Currently, systematic studies simultaneously probing the effects of ion irradiation and elevated temperature on GaN’s mechanical properties are lacking. Such investigations are pivotal for advancing the development of high-performance GaN-based devices.

In this work, systematic nanoindentation measurements from room temperature (RT) to 300 °C have been performed on ion-irradiated and unirradiated GaN single crystals. The influence of ion irradiation and temperature variations on multiple aspects, including the pop-in phenomena, material piling-up, indentation size effect, and fracture behaviors, is explored. The hardness and Young’s modulus of all samples are determined. In contrast to face-centered cubic metals like Al and Cu, which exhibit negligible Peierls stress, GaN single crystals exhibit a Peierls stress in the range of a few gigapascals, with a decreasing trend as temperature rises. Additionally, it is shown that irradiation embrittlement effects are significant in GaN single crystals. All samples show no creep behaviors at the tested temperature and irradiation levels. This work advances our understanding of the combined impacts of irradiation and temperature on the mechanical properties of GaN single crystals, and might aid the rational design of GaN-based transistors, lasers, and sensors.

## 2. Materials and Methods

### 2.1. Sample Preparation

Free-standing polar (c-plane) GaN single crystal samples, provided by Suzhou Nanowin Science and Technology Co., Ltd. (Suzhou, China), are prepared by hydride vapor phase epitaxy (HVPE). Each sample has dimensions of 10 mm × 10.5 mm, a thickness of 350 ± 25 μm, and exhibits a surface roughness smaller than 0.2 nm. Values of the sample thickness and surface roughness are provided by the company.

**Figure 1:**
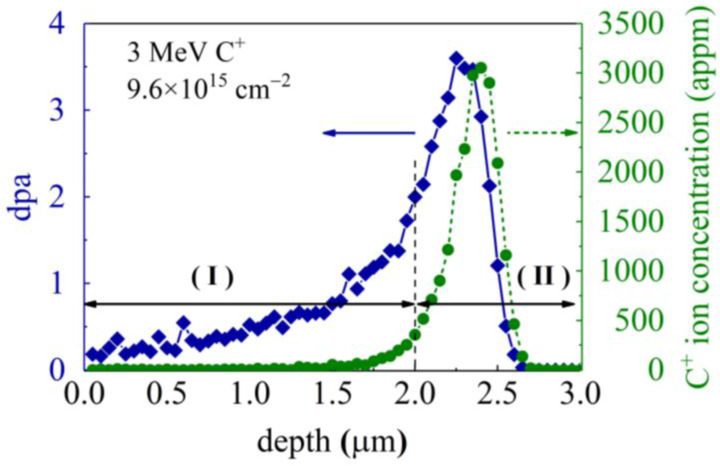
C^+^ ion concentration and dpa profiles determined using SRIM calculations for GaN implanted with C^+^ at 3 MeV with an ion fluence of 9.6 × 10^15^/cm^2^. The irradiation-induced damage extends to a depth of approximately 3 μm, with the peak damage occurring at a depth of about 2.25 μm beneath the sample surface. appm, atomic parts per million.

3 MeV C^+^ ion irradiation of the GaN single crystal samples is conducted using a 1.7 MV tandem ion accelerator at RT with a fluence of 9.6 × 10^15^ ions/cm^2^. The flux of C^+^ ion is controlled at 1 × 10^12^ ions/(cm^2^·s) to minimize significant target heating. The implanted carbon distribution and corresponding displacements-per-atom (dpa) profile are determined using SRIM (stopping and range of ions in matter) simulations [19] with “Quick” Kinchin-Pease option [20] (Figure 1). The dpa versus damage depth profile can be roughly divided into two regions: in region I, the dpa value or damage level gradually increases with depth, while region II displays a rapid variation, signifying a significantly non-uniform damage distribution. To mitigate the influence of this non-uniform damage distribution on the nanoindentation analysis, the indentation depth of the irradiated GaN samples is confined to below 200 nm.

### 2.2. Nanoindentation Procedure and Analysis

High-temperature nanoindentation tests on both unirradiated and ion-irradiated samples are performed using a Hysitron TI 980 Triboindenter (Bruker, Minneapolis, MN, USA) equipped with an xSol 800 heating stage [21] and integrated with a scanning probe microscopy (SPM) system. As tip-shape controls performed after tests at temperature from RT to 300 °C show no change in area function [22], in this study, the area function and machine compliance are calibrated at RT on fused quartz, following the ISO14577 standard procedure [23]. The Berkovich indenter is affixed to a low-thermal-conductivity shaft to reduce heat transfer from the surface to the transducer and prevent the oxidation or melting of the standard probe holder at high temperature [24]. The sample is sandwiched and heated using resistance heating plates. Each heating plate is equipped with a thermocouple to measure and control the testing temperature and is also equipped with a water coolant and environmental gas flow system. The heating rate is controlled by the equipment software, with an average heating rate of 20 °C/min. The sample is first heated to a selected target temperature and a holding time of around ten minutes is required to reach thermal equilibrium due to the excellent thermal stability of the xSol heating stage. In the high-temperature nanoindentation experiments, high-purity nitrogen gas (>99.9992%) is used to shield the GaN sample surface from oxidation. To maintain thermal equilibrium and minimize thermal drift, the probe is positioned 10 μm above the sample surface before each indentation test.

Nanoindentation tests of unirradiated and ion-irradiated samples are carried out at RT, 100 °C, 200 °C, and 300 °C with a load-controlled partial unloading/cyclical mode. The load function contains a total of ten partial unloading cycles, and each cycle comprises three consecutive segments of 0.4 s loading, 0.1 s holding, and 0.5 s unloading. The minimum loading force, maximum loading force, cyclic loading exponent, and unloading fraction are 3 mN, 12 mN, 1, and 0.5, respectively. At least ten indents are performed on individual samples at each temperature, and average values of the hardness *H* and reduced Young’s modulus E* are calculated. The space separation between indents is large enough to avoid plastic zone interaction. Once load–displacement curves are obtained, the hardness *H* and reduced Young’s modulus E* at different values of temperature and indentation depth are determined from the unloading part using the Oliver and Pharr method as [25]

(1) $$H=\frac{P}{A},$$

(2) $$E*=\frac{\sqrt{\pi }}{2\beta }\frac{S}{\sqrt{A}},$$

where *P* is the indenter load, *A* is the projected area of contact whose value depends on the indenter geometry and contact depth, the indenter geometry shape factor *β* is taken as 1.034 for Berkovich indenter [11,26], and $S={\left.dP/dh\right|}_{h=h_{max}}$ is the contact stiffness. The indenter area function *A*(*h*_c_) takes the form [25]

(3) $$A=C_{0}h_{c}^{2}+C_{1}h_{c}+C_{2}h_{c}^{1/2}+C_{3}h_{c}^{1/4}+C_{4}h_{c}^{1/8}+\ldots ,$$

where *h*_c_ is the depth of circle of contact measured from maximum depth *h*_max_ (contact depth). Before the nanoindentation measurements, indentations are first performed on a fused silica sample to determine values of coefficients *C*_0_ (=24.5 for an ideal Berkovich tip), *C*_1_, *C*_2_, ⋯, etc. The constants *C_k_* (*k* = 1, 2, ⋯) are coefficients determined by fitting measured *A* versus *h*_c_ data, and their associated terms in Equation (3) depict deviations from the ideal Berkovich geometry due to blunting at the tip. Once E* is determined, the Young’s modulus *E*_GaN_ of GaN samples could be obtained from

(4) $$\frac{1}{E*}=\frac{1-\nu_{i}^{2}}{E_{i}}+\frac{1-\nu_{GaN}^{2}}{E_{GaN}},$$

where *ν*_i_ and *E*_i_ denote the Poisson ratio and Young’s modulus of the indenter tip, respectively, and *ν*_GaN_ is the Poisson ratio of the GaN samples, which is taken as 0.25 [27]. For diamond indenters used here, *E*_i_ = 1141 GPa and *ν*_i_ = 0.07. 

The Berkovich diamond probe is also used as an SPM tip to in situ measure the sample surface topography. The SPM made it possible to examine and measure the pile-ups. The presence of pile-ups at the periphery of the contact impression would affect the evaluation of contact area and the subsequent hardness. By approximating the pile-up contact perimeter as a semi-ellipse, the true contact area *A*_s_ of the indentation for irradiated GaN single crystals with evident pile-ups is given by [28]

*A*_s_ = *A* + 5.915*h*_c_∑*a_j_*  (*j* = 1, 2, 3), (5)

where *A* and *h*_c_ are obtained using the Oliver–Pharr method [25], and *a_j_* is the horizontal distance between the pile-up contact perimeter to the edge of the indentation, with *j* = 1, 2, and 3 denoting the three semi-elliptical projected pile-up lobes. Replacing the contact area *A* in Equation (3), the true contact area *A*_s_ given by Equation (5) is used to calculate the hardness of the irradiated GaN single crystals. 

To study the effects of irradiation and temperature on the fracture behaviors of GaN single crystals, the load-controlled indents are applied with larger loads, involving a trapezoidal loading sequence: 5 s linear loading, 2 s holding segment at the peak load, and 5 s linear unloading. The maximum load is set as 3 N, and each temperature condition is tested with a minimum of three indents on individual samples. The crack morphology is examined promptly after the nanoindentation tests. 

Nanoscale Dynamic Mechanical Analysis (nanoDMA) is a technique used to analyze the dynamic mechanical properties of materials at the nanoscale, encompassing attributes like energy dissipation and time-dependent deformation characteristics. To study the influence of irradiation and temperature on the creep behaviors of GaN single crystals, nanoDMA tests are carried out. These tests employ specific parameters: a peak force of 12 mN, a frequency of 220 Hz, a load amplitude of 120 µN, and a total test time of 600 s. At least three indents are performed on individual samples at each temperature. Once the indentation experiments at a selected temperature are finished, the temperature is increased to the next selected value. The time interval between tests at different temperatures is within one hour.

## 3. Results

### 3.1. Effects of Temperature and Ion Irradiation on Pop-In Load

**Figure 2:**
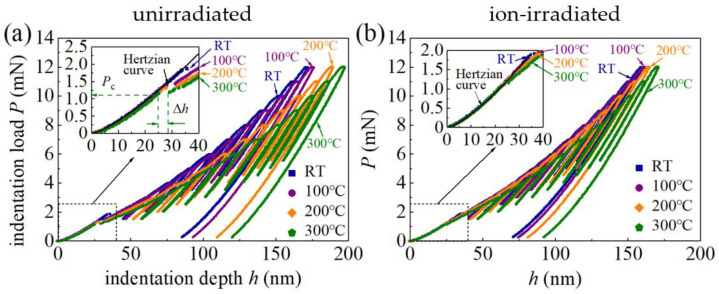
Partial unloading/cyclical curves of (**a**) the unirradiated and (**b**) ion-irradiated GaN single crystal samples for indents performed at RT, 100 °C, 200 °C, and 300 °C, with a force range from 3 mN to 12 mN. Insets, magnified view of first pop-in discontinuities.

Figure 2 shows the typical partial unloading/cyclical load–penetration depth (*P*–*h*) curves performed at RT, 100 °C, 200 °C, and 300 °C. The range of the indentation force is from 3 mN to 12 mN. Under a maximum loading force of 12 mN, the unirradiated GaN single crystals exhibit a notable increase in indentation depth, with *h* progressing from 172.22 nm at RT to 200 nm at 300 °C, signifying a 16.1% increment. In contrast, the ion-irradiated GaN single crystals demonstrate a more modest rise in *h*, registering a 7.5% increase, from 158.88 nm at RT to 170.82 nm at 300 °C, under *P* = 12 mN. Since the maximum indentation depth *h*_max_ is around 200 nm, significantly smaller than the thickness of the damage region I as depicted in Figure 1, the substrate effect could be neglected. 

**Figure 3:**
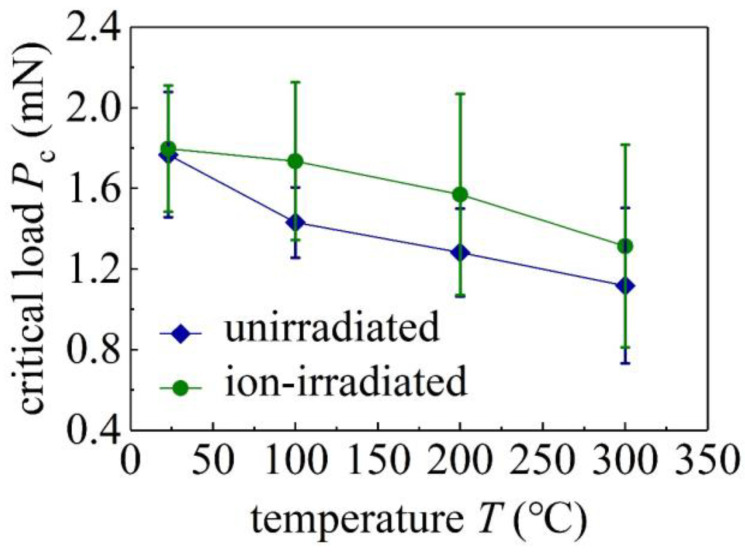
Critical loads *P*_c_ of unirradiated and ion-irradiated GaN single crystals at different temperature *T*. Error bars, standard deviations.

The pop-in phenomena (sudden displacement jump) in *P*–*h* curves are observed at shallow indentation depths (Figure 2). Similar pop-in phenomena have been observed in nanoindentation of GaN single crystals [13,29,30], GaN thin films on substrates [31], and as well as many other brittle semiconductor materials (e.g., Si, InP, ZnO, and GaAs) [32]. The first pop-in events in GaN single crystals under nanoindentation are associated with the critical transition from pure elastic behavior to plastic deformation [14] and are usually attributed to dislocation nucleation and propagation during loading [32]. This is reflected in *P*–*h* curves in Figure 2, which are well characterized by the agreement with and deviation from the Hertzian relationship before and after the first pop-in phenomenon. The load at the occurrence of the first pop-in event is recorded as the critical load *P*_c_, and the sudden displacement jump Δ*h* is recorded as the displacement excursion (Figure 2a). The average critical loads *P*_c_ of the unirradiated and ion-irradiated samples as functions of temperature are shown in Figure 3, and the error bars represent the standard deviations of at least ten measurements per temperature. These values are included in Table 1.

The first pop-in events in GaN single crystals are primarily associated with the sudden nucleation and propagation of dislocations gliding along the most active slip systems [31,33]. Defects in the disordered layer of the ion-irradiated GaN single crystals may act as dislocation pinning points [10]. The extent to which the dislocations are pinned may affect the critical load *P*_c_. Figure 3 shows that, at the same temperature, the average value of *P*_c_ of the ion-irradiated samples is larger than that of the unirradiated samples, and values of *P*_c_ of both the unirradiated and ion-irradiated samples decrease as the temperature *T* increases. These results are consistent with the nanoindentation of ion-bombarded modified wurtzite GaN films [10], showing that the elastic behaviors of ion-irradiated GaN samples extend to higher loads than unirradiated GaN samples. As the nucleation and propagation of dislocations are stress-assisted and thermally activated, the statistical distribution of the pop-in phenomenon is affected by both temperature and loading rate. Since the loading rate remains constant in this study, the reduction in the average critical load *P*_c_ for the unirradiated or ion-irradiated samples is mainly caused by the increasing temperature.

### 3.2. Effects of Temperature and Ion Irradiation on Young’s Modulus

**Figure 4:**
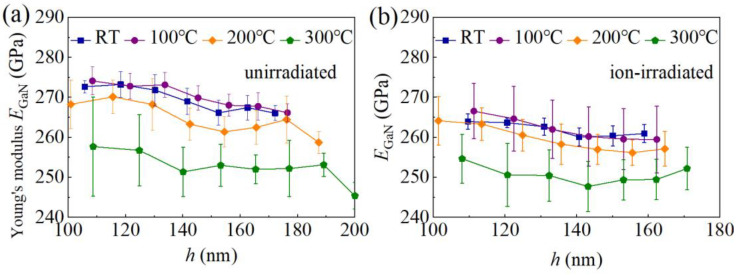
Young’s moduli *E*_GaN_ of unirradiated (**a**) and ion-irradiated (**b**) GaN single crystal samples versus the indentation depth at different temperatures. Error bars, standard deviations.

**Figure 5:**
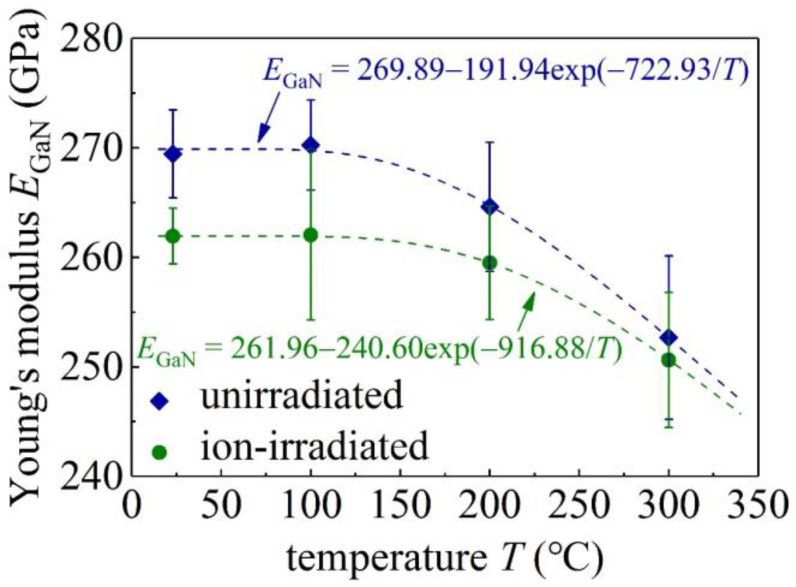
Average Young’s moduli *E*_GaN_ in Figure 4 at different values of *T*. Error bars, standard deviations.

Figure 4 shows Young’s moduli *E*_GaN_ of both unirradiated and ion-irradiated GaN single crystals, exhibiting no clear indentation size effects, which further ensures the accuracy of the measured hardness shown below. The average values of *E*_GaN_ of the unirradiated and ion-irradiated GaN samples at different values of *T* are shown in Figure 5 and are also listed in Table 1. As the temperature *T* increases from RT to 100 °C, *E*_GaN_ remains at the same value. In contrast, *E*_GaN_ slightly decreases as *T* increases from 100 °C to 300 °C. At RT, the *E*_GaN_ of the unirradiated GaN samples is 269.5 ± 3.1 GPa (Table 1), in good agreement with reported experimental values [12,27,29], and a there is a reduction of 2.8% upon the C ion irradiation. The ion irradiation-induced slight reduction in Young’s modulus has also been observed in the nanoindentation of SiC samples with a Young’s modulus reduction of less than 5% after ion irradiation [4]. Such a slight reduction in Young’s modulus is attributed to an increase in the atomic spacing caused by irradiation [4].

### 3.3. Effects of Temperature and Ion Irradiation on Hardness

**Figure 6:**
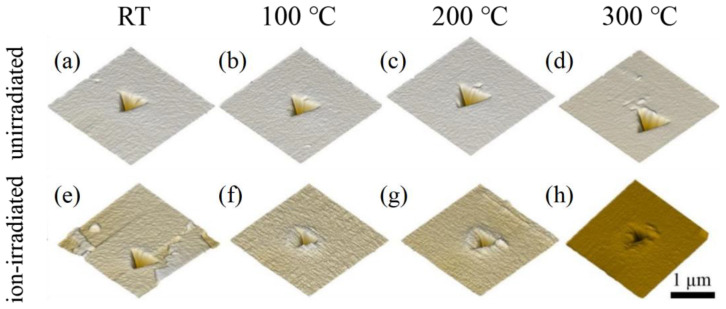
Images of surface topography of the indentation sites for unirradiated (**a**–**d**) and ion-irradiated (**e**–**h**) GaN single crystals at RT (**a**,**e**), 100 °C (**b**,**f**), 200 °C (**c**,**g**), and 300 °C (**d**,**h**).

Evident pile-ups of material around the indentation could be observed for the ion-irradiated GaN single crystals in comparison with the unirradiated GaN samples (Figure 6), consistent with reported nanoindentation results of GaN thin films upon irradiation with swift heavy uranium ions [12]. In addition, all samples show no creep behavior at the tested temperatures.

**Figure 7:**
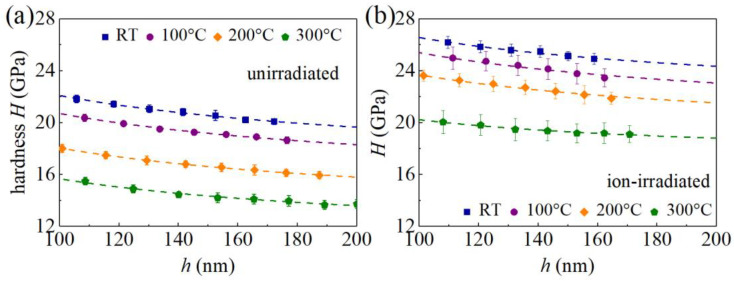
Profiles of the hardness *H* versus indentation depth *h* for unirradiated (**a**) and ion-irradiated (**b**) GaN single crystals at different temperatures. Symbols, experimental values; dashed curves, fitting results from Equation (7); error bars, standard deviations.

Figure 7 shows the hardness *H* of GaN samples as a function of the indentation depth *h*. As *h* increases, *H* decreases, a typical feature of the indentation size effect. As shown in Figure 7, the hardness *H* of the unirradiated GaN single crystals at RT for indentation depth *h* = 172 nm is 20.1 GPa, which is consistent with the hardness value 20 ± 1 GPa of unirradiated GaN thin films measured by nanoindentation at an indentation depth of 200 nm reported in the literature [12], and is also consistent with the hardness value 19.2 ± 0.2 GPa of unirradiated GaN single crystals measured by nanoindentation *h* = 160 nm in our recent studies [13]. The hardness of GaN samples measured in the literature may be different due to the different quality of the GaN samples. Considering the generation of geometrically necessary dislocations (GNDs) underneath the indenter, the classical Nix–Gao model predicts the depth-dependent hardness *H* as [34]

*H*/*H*_0_ = (1 + h*/*h*)^1/2^, (6)

where *H*_0_ is the hardness without the presence of GNDs and h* = 40.5*bα*^2^tan^2^*θ*(*μ*/*H*_0_)^2^ is a length characterizing the depth dependence of hardness, depending on the magnitude *b* of the Burgers vector and shear modulus *μ* of the material, coefficient *α*, the angle *θ* between the surface of the specimen plane and Berkovich indenter, and *H*_0_. In the Nix–Gao model, the effect of intrinsic lattice resistance or Peierls stress on the hardness is ignored. As the Peierls stress is very small for Al and Cu, the Nix–Gao model is well justified for these face-centered cubic metals [35,36,37]. 

**Figure 8:**
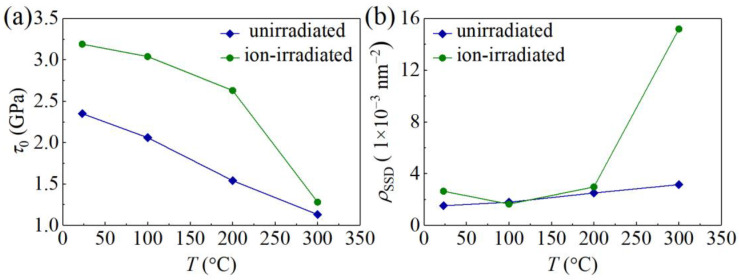
(**a**) *τ*_0_ and (**b**) *ρ*_SSD_ of unirradiated and ion-irradiated GaN samples at different values of temperature *T*.

For GaN single crystals, the Peierls stresses of dislocations of different slip systems are of at least 0.3373 GPa, and the maximum value could reach 27.8631 GPa [38]. Therefore, the Peierls stresses of GaN cannot be ignored. Based on the Nix–Gao model, while taking into account the Peierls stress *τ*_0_, the hardness *H* as a function of the indentation depth *h* is given as [39]

(7) $$H=MC\tau_{0}+MC\alpha \mu b\sqrt{\rho_{SSD}+\rho_{GND}(h)},$$

where the Taylor factor *M* is taken as $\sqrt{3}$, the Tabor factor *C* is taken as 3, *α* = 0.5, the shear modulus *μ* = *E*_GaN_/[2(1 + *ν*_GaN_)] with *ν*_GaN_ = 0.25 [27], *b* = 3.191 Å [40], *ρ*_SSD_ is the density of statistically stored dislocations (SSDs), and the GND density is *ρ*_GND_(*h*) = 3tan^2^*θ*/(2*bh*) with *θ* = 24.73°. Fitting experimental data in Figure 7 with Equation (7), values of *τ*_0_ and *ρ*_SSD_ at different values of *T* are determined and are shown in Figure 8. For unirradiated GaN samples at RT, *τ*_0_ = 2.35 GPa, falling in a range from 0.34 GPa to 27.86 GPa based on MD simulations [40]. As *T* increases, *τ*_0_ of the unirradiated GaN samples decreases to 1.13 GPa at 300 °C, and *τ*_0_ of the ion-irradiated GaN samples decreases from 3.19 GPa at RT to 1.28 GPa at 300 °C. The Peierls stress *τ*_0_ becomes larger after irradiation due to the pinning effect induced by the irradiation-induced defects.

The classical indentation hardness or intrinsic hardness *H*_0_ without the presence of GNDs is

(8) $$H_{0}=MC\tau_{0}+MC\alpha \mu b\sqrt{\rho_{SSD}}.$$

**Figure 9:**
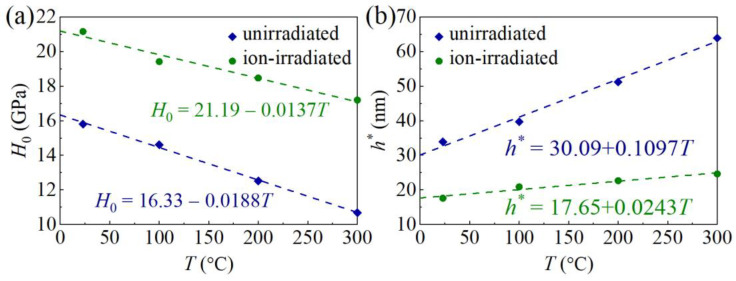
Effects of temperature and irradiation on *H*_0_ (**a**) and h* (**b**). Dashed lines, linear fitting.

Values of *H*_0_ of the unirradiated and ion-irradiated GaN samples at different values of *T* are listed in Table 1 and the corresponding *H*_0_–*T* curves are plotted in Figure 9a. It is shown that the *H*_0_ of the ion-irradiated GaN single crystals is larger than that of unirradiated GaN sample at the same temperature, indicating an ion irradiation hardening effect. This phenomenon has been consistently observed in GaN subjected to ion irradiation with O^+^, N^+^, Mg^+^, and Si^+^ ions [8,9]. The increased hardness is attributed to irradiation-induced defects that effectively immobilize dislocations and impede plastic deformation. Nanoindentation tests on ion-irradiated GaN single crystals reveal the concurrent presence of both the indentation size effect and irradiation hardening. 

The intrinsic hardness *H*_0_ linearly decreases with increasing *T* (Figure 9a). For the unirradiated GaN single crystals, *H*_0_ = 16.33 − 0.0188*T*, a reduction of 32.4% from RT to 300 °C; for the ion-irradiated GaN single crystals, *H*_0_ = 21.19 − 0.0137*T*, a reduction of 18.7% from RT to 300 °C. The linear decrease in intrinsic hardness *H*_0_ of the unirradiated GaN single crystals with increasing temperature *T* aligns with reported MD simulation results [18]. Due to the relatively high Peierls stress *τ*_0_ in GaN, the dislocations move slowly, and the dislocation cross-slip enhances the likelihood of forming dislocation networks [38,41]. As *T* rises, increased thermal energy facilitates thermal activation over the Peierls barrier, leading to reduced lattice friction or Peierls stress for dislocation motion. According to Equation (8), the decreasing hardness of GaN with increasing *T* might be linked to the diminishing Peierls stress *τ*_0_ [15,39]. Comparable linear decreases in intrinsic hardness from RT to 400 °C have been observed in various materials, including tungsten, tungsten carbide, diamond, and boron nitride [42,43]. However, over a broader temperature range from RT to 1000 °C, a nonlinear hardness–temperature relationship becomes apparent [42]. Further mechanistic studies are necessary to unveil the intricacies of the hardness–temperature relationship.

**Figure 10:**
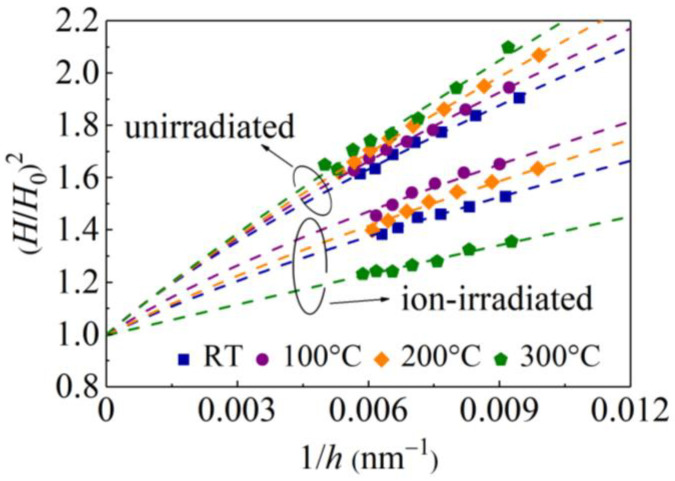
Normalized hardness (*H*/*H*_0_)^2^ versus the inverse indentation depth 1/*h* for unirradiated and ion-irradiated GaN single crystals at different temperatures. Symbols, experimental values; dashed curves represent fitting results from Equation (9).

A combination of Equations (7) and (8) leads to [39]

(9) $$\frac{H}{H_{0}}=\frac{MC\tau_{0}}{H_{0}}+\sqrt{{\left(1-\frac{MC\tau_{0}}{H_{0}}\right)}^{2}+\frac{h*}{h}},$$

where h* in Equation (9) has the same expression as that in the Nix–Gao model, but it also depends on the Peierls stress via *H*_0_. Fitting the experimental data in Figure 9 with Equation (9), values of h* at different *T* can be obtained (Figure 9b and Table 1). Then, the (*H*/*H*_0_)^2^ − 1/*h* relationship can be obtained (Figure 10). 

### 3.4. Effects of Temperature and Ion Irradiation on Fracture

**Figure 11:**
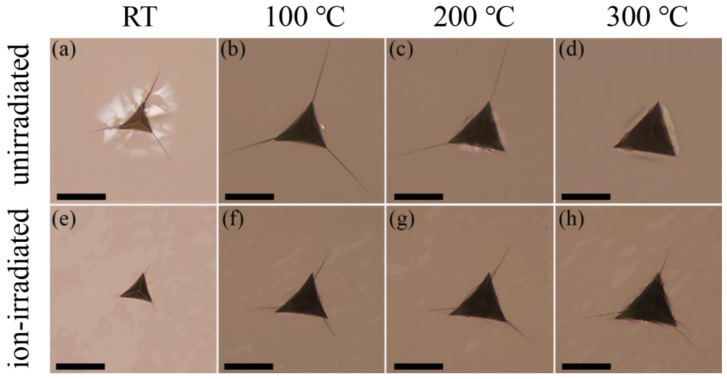
Optical micrographs of residual contact impression with indentation-induced cracking in unirradiated (**a**–**d**) and ion-irradiated (**e**–**h**) GaN single crystals at a maximum load of 3 N for RT (**a**,**e**), 100 °C (**b**,**f**), 200 °C (**c**,**g**), and 300 °C (**d**,**h**). Scale bars, 20 µm.

Figure 11 shows optical micrographs of residual impression with indentation-induced cracking in unirradiated and ion-irradiated GaN single crystals at a maximum load of 3 N for all tested temperatures. At RT, radial cracks emanate from three corners of the Berkovich indentation print, and the radial cracks in the ion-irradiated GaN single crystals are significantly shorter than these in the unirradiated GaN single crystals (Figure 11a,e). Notably, the residual impression of ion-irradiated GaN single crystals at RT is smaller than that of the unirradiated samples. Three-dimensional MD simulation results of c-plane monocrystalline GaN under nanoindentation show that the nucleation and propagation of dislocations in the main slip systems are promoted to intensify the plastic deformation of the GaN crystal [18]. As shown in the results of the high-temperature nanoindentation tests (Figure 11b–d), the residual impression in the unirradiated GaN single crystals becomes larger as temperature increases. In the unirradiated GaN single crystals, the radial cracks emanating from three corners of the Berkovich indentation print at 100 °C (Figure 11b) become larger than those at RT (Figure 11a). However, the brightening areas in unirradiated GaN single crystals (Figure 11a) caused by specular reflection at the lateral subsurface cracks disappear at high temperature (Figure 11b–d). The length of the radial cracks of unirradiated GaN single crystals at 200 °C (Figure 11c) changes little compared with that at 100 °C. However, a radial crack from one corner of the Berkovich indentation print of unirradiated GaN single crystals at 200 °C (Figure 11c) disappears. Further, all the radial cracks of unirradiated GaN single crystals at 300 °C disappear. As the temperature increases, the brittleness of unirradiated GaN single crystals tend to weaken. For ion-irradiated GaN single crystals at high temperature (Figure 11f–h), as the temperature increases, the residual impression becomes slightly larger, the radial cracks from the corners seem to be independent of temperature, and erratically shaped small radial cracks begin to appear both from the corners and edges of the Berkovich indentation print.

It has been demonstrated that irradiation-induced residual stress can inhibit crack formation during nanoindentation [44]. However, with an increase in temperature, these residual stresses tend to gradually dissipate. Consequently, a noticeable trend emerges, where the brittleness of ion-irradiated and unirradiated GaN single crystals exhibits opposite behaviors as temperature rises. Specifically, at RT, radial and lateral cracks in ion-irradiated GaN single crystals are notably smaller in comparison to unirradiated samples (Figure 11a,e). In contrast, at 300 °C, unirradiated GaN single crystals exhibit no radial or lateral cracks, whereas radial cracks originating from the corners and edges of ion-irradiated GaN samples remain apparent (Figure 11d,h). This observation, while excluding the influence of residual stress, suggests that irradiation elevates the ductile-to-brittle transition temperature of GaN single crystals, indicating the presence of irradiation-induced embrittlement effects. Furthermore, as temperature increases, it becomes evident that the residual impressions on both unirradiated and ion-irradiated GaN single crystals gradually enlarge, indicating enhanced plasticity at higher temperatures.

## 4. Conclusions

Systematic experimental investigations on the effects of C ion irradiation and temperature on the hardness, Young’s modulus, and fracture of GaN single crystals under nanoindentation have been performed. It is found that the critical indentation load for the occurrence of the pop-in phenomenon decreases as the temperature increases. Moreover, ion-irradiated GaN samples have a larger critical load for pop-in in comparison with unirradiated samples, suggesting that the dislocation nucleation or propagation is suppressed by the irradiation-induced defects. For the hardness of both unirradiated and ion-irradiated GaN samples, the typical feature of the indentation size effect is observed, that is, the hardness decreases with the increasing indentation depth. In contrast, Young’s moduli of both unirradiated and ion-irradiated GaN single crystals do not exhibit a clear size effect. In addition, the macroscopic hardness decreases as the temperature increases, attributed to the decreasing Peierls stress with increasing temperature. As irradiation-induced defects could pin dislocations and prohibit the plastic deformation, the ion-irradiated GaN single crystals have a larger macroscopic hardness than that of unirradiated samples. Optical micrographs of indentation-induced fracture further indicate irradiation embrittlement effects. No creep behaviors are observed at tested temperatures. Our studies aid understanding the mechanical effects of irradiation damage on GaN single crystals and might have broad implications in improving the service life of GaN-based devices.

## Figures and Tables

**Table 1:** Measured mechanical properties of unirradiated and irradiated GaN single crystals by nanoindentation at different temperature *T*.

*T* (°C)	Unirradiated				Ion-Irradiated			
	*P*_c_ (mN)	*E*_GaN_ (GPa)	*H*_0_ (GPa)	h* (nm)	*P*_c_ (mN)	*E*_GaN_ (GPa)	*H*_0_ (GPa)	h* (nm)
RT	1.77 ± 0.31	269.5 ± 3.1	15.81	33.89	1.80 ± 0.31	261.9 ± 1.7	21.16	17.58
100	1.43 ± 0.17	270.2 ± 3.1	14.60	39.70	1.73 ± 0.39	262.1 ± 2.9	19.42	20.87
200	1.28 ± 0.22	264.6 ± 3.9	12.52	51.18	1.57 ± 0.50	259.5 ± 3.2	18.47	22.65
300	1.12 ± 0.39	252.7 ± 3.7	10.68	63.90	1.42 ± 0.50	250.6 ± 2.2	17.19	24.62

## Data Availability

Data are contained within the article.
